# Biosensor-Based Approach Identifies Four Distinct Calmodulin-Binding Domains in the G Protein-Coupled Estrogen Receptor 1

**DOI:** 10.1371/journal.pone.0089669

**Published:** 2014-02-21

**Authors:** Quang-Kim Tran, Mark VerMeer

**Affiliations:** Department of Physiology & Pharmacology, Des Moines University Osteopathic Medical Center, Des Moines, Iowa, United States of America; University of Oldenburg, Germany

## Abstract

The G protein-coupled estrogen receptor 1 (GPER) has been demonstrated to participate in many cellular functions, but its regulatory inputs are not clearly understood. Here we describe a new approach that identifies GPER as a calmodulin-binding protein, locates interaction sites, and characterizes their binding properties. GPER coimmunoprecipitates with calmodulin in primary vascular smooth muscle cells under resting conditions, which is enhanced upon acute treatment with either specific ligands or a Ca^2+^-elevating agent. To confirm direct interaction and locate the calmodulin-binding domain(s), we designed a series of FRET biosensors that consist of enhanced cyan and yellow fluorescent proteins flanking each of GPER’s submembrane domains (SMDs). Responses of these biosensors showed that all four submembrane domains directly bind calmodulin. Modifications of biosensor linker identified domains that display the strongest calmodulin-binding affinities and largest biosensor dynamics, including a.a. 83–93, 150–175, 242–259, 330–351, corresponding respectively to SMDs 1, 2, 3, and the juxta-membranous section of SMD4. These biosensors bind calmodulin in a strictly Ca^2+^-dependent fashion and with disparate affinities in the order SMD2>SMD4>SMD3>SMD1, apparent K*_d_* values being 0.44±0.03, 1.40±0.16, 8.01±0.29, and 136.62±6.56 µM, respectively. Interestingly, simultaneous determinations of biosensor responses and suitable Ca^2+^ indicators identified separate Ca^2+^ sensitivities for their interactions with calmodulin. SMD1-CaM complexes display a biphasic Ca^2+^ response, representing two distinct species (SMD1 sp1 and SMD1 sp2) with drastically different Ca^2+^ sensitivities. The Ca^2+^ sensitivities of CaM-SMDs interactions follow the order SMD1sp1>SMD4>SMD2>SMD1sp2>SMD3, EC_50_(Ca^2+^) values being 0.13±0.02, 0.75±0.05, 2.38±0.13, 3.71±0.13, and 5.15±0.25 µM, respectively. These data indicate that calmodulin may regulate GPER-dependent signaling at the receptor level through multiple interaction sites. FRET biosensors represent a simple method to identify unknown calmodulin-binding domains in G protein-coupled receptors and to quantitatively assess binding properties.

## Introduction

Plasma estrogen concentrations are closely related to cardiovascular health. Along with menopause comes a substantial increase in the risk of cardiovascular diseases [1], [2]. Estrogen modulates gene expression, growth, development and immune responses, and has many cardiovascular protective effects. The actions of estrogen are extensive and include effects that are both dependent and independent of transcriptional activities [3], [4]. The mechanisms underlying these effects are still far from being completely understood [5], [6]. Indeed, two classical estrogen receptors, ERα and ERβ, which function as transcriptional factors that bind estrogen responsive elements in promoters of target genes [3], were thought to be totally responsible for estrogen’s effects. However, a novel G protein-coupled receptor, GPR30, was cloned around 1997 as an orphan receptor [7]–[11], and was demonstrated to be an estrogen receptor in 2005 [12], [13]. It was subsequently termed G protein-coupled estrogen receptor 1 (GPER) by International Union of Basic and Clinical Pharmacology (IUPHAR). GPER has since been shown to be involved in many cellular activities, including Ca^2+^ mobilization [13], [14], cAMP production [12], [14], activation of protein kinases [13], [15], and activation of transcription [16]–[19]. Clarifying the regulation of GPER and related pathways will enhance our knowledge of how estrogen works and provide grounds to target estrogen receptor subtypes for preventive and therapeutic purposes.

Calmodulin (CaM) is a highly conserved 148-a.a. protein that contains four EF-hand Ca^2+^-binding motifs. Ca^2+^-bound CaM has a dumbbell conformation with two EF-hand motifs on either end connected by a central helix. This central helix functions as a tether that is bent upon target interaction while the two lobes exert concerted effects [20]. Ca^2+^ binding exposes hydrophobic patches, promoting CaM’s interaction with its target proteins. In this fashion, CaM is the ubiquitous transducer of cellular Ca^2+^ signals and is involved in virtually all aspects of cellular functions due to its interaction with and requirement for the activities of hundreds of target proteins [21]–[23]. The expression of CaM fluctuates with cell cycle [24], but has been shown to be insufficient to saturate all targets’ binding sites in a significant number of cell types, including vascular endothelial cells [25], [26], smooth muscle cells [27], and cardiomyocytes [28], [29]. This insufficiency of CaM has been demonstrated to generate functional coupling among its targets due simply to competition for CaM [25], [26], and suggests that factors controlling CaM expression and dynamics can vastly alter cellular functions.

G protein-coupled receptors (GPCRs) represent a superfamily of cell surface proteins that convey extracellular inputs to vast changes in cellular functions via dynamic associations with heterotrimeric G proteins and numerous other partners at their submembrane domains. Recently, CaM has been demonstrated to interact with a number of G protein-coupled receptors (GPCRs), such as the metabotropic glutamate receptors mGluR1 and mGluR5 [30], [31], the opioid μ receptor [32], the parathyroid hormone receptor 1 [33], the 5-HT(2C) and 5-HT(1A) receptors [34], [35], the D_2_ dopamine receptor [36], and the angiotensin II receptor type 1A [37], [38]. In the case of the 5HT(1A) receptor, CaM binding to the third submembrane domain alters the receptor’s phosphorylation or interaction with G protein subunit(s) [35], while for the 5-HT(2C) receptor, CaM binding to the C terminal tail has been demonstrated to be important for β-arrestin recruitment and for receptor-operated extracellular signal-regulated kinase [39]. Nevertheless, the roles of CaM in GPCR biology at the receptor level is still not entirely clear. Part of the reason for this is the lack of an approach to exhaustively identify all interaction sites for CaM on a GPCR. For example, a CaM-binding domain in the juxtamembrane region of the cytoplasmic tail of the angiotensin II receptor type 1A was first identified in 1999 using a peptide deduced from a sequence-based comparison with known CaM-binding motifs [37]. More than a decade later, another CaM-binding domain in the third submembrane domain in the same receptor was recently identified using a similar approach [38]. In addition, while the interactions between CaM and these GPCRs are Ca^2+^-dependent, information regarding the specific Ca^2+^ sensitivities of these interactions is lacking. Knowledge of the Ca^2+^ sensitivities of GPCR-CaM interactions will be of value in determining the roles of CaM interaction with each submembrane domain at different physiological scenarios in cells.

With respect to estrogen biology, it is completely unknown if CaM plays a role GPER-dependent signaling. We have begun to test the idea that CaM is directly involved in GPER-dependent signaling in the vasculature at both the receptor level and downstream effectors. In this paper, we describe a new approach to identify GPER as a novel CaM-binding protein, locate all of its CaM-binding domains, and determine their binding characteristics including K*_d_* and Ca^2+^ sensitivities. Using FRET-based biosensors designed to scan all submembrane domains of GPER, we identified up to four binding sites located each in submembrane domains 1, 2, 3 and 4 of GPER that have distinct binding affinities for CaM. In addition, we simultaneously incorporated these biosensors and suitable Ca^2+^ indicators in single assays to precisely determine the Ca^2+^ sensitivities of the interactions between CaM and these different domains in GPER. This approach in turn revealed quite distinct sensitivities to Ca^2+^ of the interactions between CaM and GPER’s submembrane domains. Interestingly, submembrane domain 1 interacts with CaM with biphasic Ca^2+^ dependency, representing two distinct populations of complexes formed at drastically different free Ca^2+^ values that range from cytoplasmic resting levels to those that occur during Ca^2+^ entry. The results demonstrate that FRET biosensor-based screening is an effective and efficient approach to identify unknown CaM-binding domains in GPCRs that allows for highly quantitative assessment of their binding properties.

## Materials and Methods

### Ethics Statement

The protocol to isolate primary vascular smooth muscle cells from porcine aortas (obtained commercially from a local slaughter house) was approved by the Des Moines University Institutional Biosafety Committee.

### Cell Isolation and Culture

Descending segments of porcine thoracic aortas were freshly collected from a local slaughter house. Primary vascular smooth muscle cells (VSMCs) were isolated from these aortas using a modification of the methods published by Bolzon [40], Ulrich-Merzenich [41], and Leik [42]. Briefly, endothelial cells were initially completely removed by a sequence of mechanical scraping, short enzyme incubation (0.02% collagenase, 0.1% papain, and 4 mM dithiothreitol) and re-scraping. After rinsing in sterile PBS, aortas were cut into rectangular strips and the luminal surface was incubated with the same enzyme mix for 90 minutes. The luminal surface was then scraped again, and cells from this enzyme treatment and scraping were grown in M-199 containing 10% fetal bovine serum and 2% penicillin/streptomycin in 90% humidified condition with 5% CO_2_ at 37°C for one week. This procedure consistently yields highly homologous populations VSMCs, verified by both morphological properties and smooth muscle α-actin immunofluorescence staining. This protocol was approved by Des Moines University Institutional Biosafety Committee.

### Co-immunoprecipitation

Co-immunoprecipitation was performed using a Pierce immunoprecipitation kit (Rockford, IL) as per manufacturer’s instructions. The use of an amino-reactive resin in this approach prevents the co-elution of the antibody heavy and light chains that may co-migrate with the bands of the proteins of interest. Typically, 10 µL of resin was conjugated with appropriate amounts of primary antibody. After appropriate treatment, cell lysis was performed on monolayer cells without trypsinization to avoid complications having to do with shear during the process. Cells were lysed on ice for 15 minutes and the lysate was centrifuged at 21,000×*g* for 5 min at 4°C. Following pre-clearing, the protein content of the eluant was determined using the BCA assay (Pierce). One milligram of total cellular proteins was then rocked with the resin-antibody conjugates for 3 hours in 1-mL columns at 4°C prior to eluting. Following electrophoresis and transfer, membranes were cut between the levels of the bait and the prey proteins prior to incubation with separate primary antibodies against CaM (EMD Millipore Corp., MA) and GPER (R&D System, MN; GeneTex Inc., Irvine CA, or Santa Cruz Biotech, CA).

### Construction of Biosensors to Identify GPER’s CaM-binding Domains

**Figure 1:**
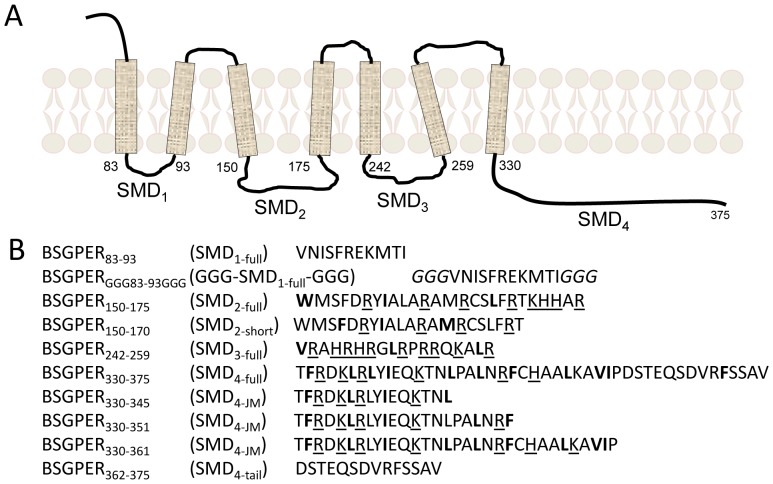
Schematic topography of human GPER (A) and sub-membrane fragments used to generate biosensors (B). SMD, sub-membrane domain. Hydrophobic residues are in bold-face type. Positively charged residues are underlined.

Biosensors to screen for CaM-binding domains in GPER were constructed based on the principle of fluorescent resonance energy transfer (FRET). Sub-membrane domains (SMDs) 1–4 from human GPER (Fig. 1A), corresponding to a.a. 83–93, 150–175, 242–259, 330–375, and shorter fragments of these domains were PCR amplified from the human GPER cDNA (Genecopoeia Inc., Rockville, MD) and inserted between enhanced variants of cyan and yellow fluorescent proteins as a donor–acceptor FRET pair. We termed these biosensors BSGPER_x_, where x denotes the amino acid numbering of the GPER insert between the fluorophores. In total, the BSGPER_x_ made were BSGPER_83–93,_ BSGPER_GGG83–93GGG_, BSGPER_150–170_, BSGPER_150–175,_ BSGPER_242–259,_ BSGPER_330–345,_ BSGPER_330–351,_ BSGPER_330–361,_ BSGPER_330–375,_ and BSGPER_362–375_ (Fig. 1B). The BSGPER_GGG83–93GGG_ construct consists of three additional glycine residues on the N- and C-terminal ends of SMD1 (see Results section). Biosensors were introduced into a bacterial expression pET vector with a 6-histidine tag at the C terminus downstream of ECFP (a gift from Dr. Anthony Persechini, University of Missouri-Kansas City).

### Expression and Purification of Biosensors

BSGPER_x_ were expressed in BL21(DE3) competent cells. An overnight culture of 5 mL was inoculated into 300 mL of 2XYT culture medium and shaken at 37°C until OD 0.6–0.8. Expression was then induced with 0.5 mM isopropyl 1-β thiogalacto-pyranoside (IPTG) and the culture was grown at 22°C for an additional 24 hrs. Expressed biosensors were then purified using standard affinity chromatography with Cb^2+^ affinity resin (Pierce). Eluant was dialyzed twice against large volumes of 50 mM Tris, 100 mM KCl, pH 7.5 at 4°C. Purified BSGPER_x_ were stored at –80°C.

### Expression and Purification of CaM

CaM was purified with a slight modification of previously published approach. A bacterial expression pET vector encoding the rat CaMI gene was transformed into BL21(DE3) E. Coli. An overnight culture of 10 mL was added to 1L of culture media and grown at 37°C until O.D. reached 0.6–0.8. Expression was induced by 1 mM IPTG. The culture was then grown for 4 hrs at 37°C. After centrifugation, pellets were resuspended in 50 mM Tris, 100 mM NaCl, pH 7.5 at room temperature. Resuspension was treated with 200 µg/mL lysozyme, protease inhibitor cocktail (0.06 units/mL) and 0.01 µL/mL PMSF for 1 hr. Cells were lysed by sonication. After centrifugation at 100,000× *g* for 30 min, 10 mM CaCl_2_ was added to the supernatant and heated to 65°C for 15 minutes. Supernatant was brought to room temperature and centrifuged again at 100,000× *g* for 30 min in the presence of 4 µL/mL DNAse and 3 mM MgCl_2_. Supernatant was loaded onto a CL-4B Phenyl Sepharose column. Columns were subjected to three long successive washes with buffer A (50 mM Tris, 1 mM CaCl_2_, pH 7.5), B (50 mM Tris, 1 mM CaCl_2_, 5 mM imidazole and 300 mM NaCl), followed by a brief wash with buffer A. CaM was eluted with 50 mM Tris, 100 mM NaCl, and 5 mM EGTA. Eluant was dialyzed twice against 50 mM Tris, 100 mM KCl, pH 7.5 and concentrated using Centriprep (Millipore) prior to use. This protocol consistently produces high yields of pure CaM.

### Binding Assay to Screen for CaM-binding Domains in GPER

BSGPER_x_ were tested *in vitro* for direct binding with purified CaM. BSGPER_x_ were mixed in titration buffer (25 mM Tris, 100 mM KCl, pH 7.5) containing 0.1 mg/mL bovine serum albumin and 1 mM CaCl_2_ in a quartz cuvette (Hellma Analytics). Small aliquots of purified CaM were added to the mixture as biosensor responses were monitored in a QuantaMaster™-40 spectrofluometer (Photon Technology International Inc.). All titrations were performed at 22°C. Direct interactions between the specified GPER fragments and CaM were identified by disruption of FRET between donor ECFP and acceptor EYFP. Biosensor fractional responses were determined by the formula
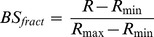
(1)where R*_min_* and R*_max_* are the ratios between emission intensities at λ475 and λ535 nm (F475/F535) when the biosensor is in unbound and maximally bound state, respectively. Fractional biosensor responses could also be determined using the emission intensity at λ475 nm (F475) or λ535 nm (F535), as follow.
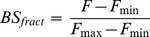
(2)where F, Fmax and Fmin represent the observed, maximal and minimal intensities at λ475 nm. Or,
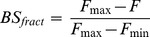
(3)where F, F, F_max_ and F_min_ represent the observed, maximal and minimal intensities at λ535 nm, as described previously [43]. Dilution factors were tightly calculated for each addition of CaM using a detailed excel spreadsheet algorithm and applied for intensity correction when the F475 or F535 values were used to derive BS_fract_; however, we determined that the use of ratiometric responses (formula (1)) negated this necessity. Precise fractional biosensor responses were plotted against titrated CaM concentrations. Apparent K_d_ values for BSGPERx-CaM interactions were obtained by fitting biosensor’s fractional responses as a function of CaM concentration to hyperbolic or quadratic binding equations [43]:



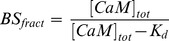
(4)

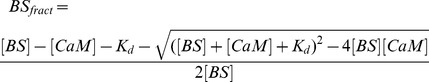
(5)where BS*_fract,_* [BS] and [CaM] are fractional response of BSGPER_x_ (equation (1)) and the total concentration of biosensor and CaM in the mixture, respectively.

Dynamic range (DR) in biosensor responses to CaM, operationally defined as the fold difference between R*_max_* and R*_min_*, reflects the conformational change that occurs upon CaM binding to the linker. This parameter was used as additional indicator of the location of the CaM-binding domain.

### Determination of Ca^2+^ Sensitivity of the Interaction between BSGPER_x_ and CaM

Ca^2+^ sensitivities of the interactions between CaM and the different submembrane domains of GPER were determined by simultaneous spectrofluorometric measurements of the responses of a suitable Ca^2+^ indicator and BSGPER_x_ in the presence of saturating CaM concentration and incremental increases in free Ca^2+^. Saturating CaM concentrations were obtained from determinations of apparent K*_d_* values of interactions between BSGPER_x_ and Ca^2+^-CaM described above. Free Ca^2+^ concentrations were determined by the responses of X-Rhod5F emission intensity at λ600 nm with excitation at λ580 nm, which were simultaneously monitored with biosensor response. Biosensor FRET emission ratio (F475/F535) following excitation at λ430 nm were concurrently monitored in a QuantaMaster-40 spectrofluorometer. Typically, starting reaction mixture contained 0.5 µM GPER biosensor, 2 µM X-Rhod5F, 0.1 mg/mL BSA, 0.25 mM Br_2_BAPTA and saturating CaM concentrations (obtained from K*_d_* titrations) in 25 mM Tris, 100 mM KCl, pH 7.5 at 22°C. In some assays with high saturating CaM concentrations, the concentration of Br2BAPTA was increased to guarantee chelation of Ca^2+^ prior to the start of Ca^2+^ titrations (see Results). In this nominally Ca^2+^-free condition, there was no interaction between CaM and BSGPER_x_. Incremental Ca^2+^ was then titrated into the reaction mix. Free Ca^2+^ values were calculated using the formula
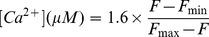
(6)where 1.6 is the *in vitro* K*_d_* value (µM) of X-Rhod5F for Ca^2+^; F_min_ and F_max_ are its fluorescence intensities measured at λ600 nm under nominally Ca^2+^-free and Ca^2+^-saturating conditions, respectively.

The Ca^2+^ sensitivities of biosensor-CaM interactions were then determined as the EC_50_(Ca^2+^) values, which were derived from fits of biosensor fractional responses (BS*_fract_*) against corresponding free Ca^2+^ concentration using the equation:
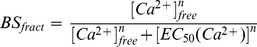
(7)where BS*_fract_* is obtained from equation (1); *n* is the Hill coefficient.

### Statistical Analysis

Data are expressed as means ± SD of at least three independent experiments. Statistical analysis was performed using Student’s *t*-test, assuming unequal variances between control and treated groups. Statistical significance was determined as P<0.05.

## Results

### Complex Formation between GPER and CaM in Primary Vascular Smooth Muscle Cells

**Figure 2:**
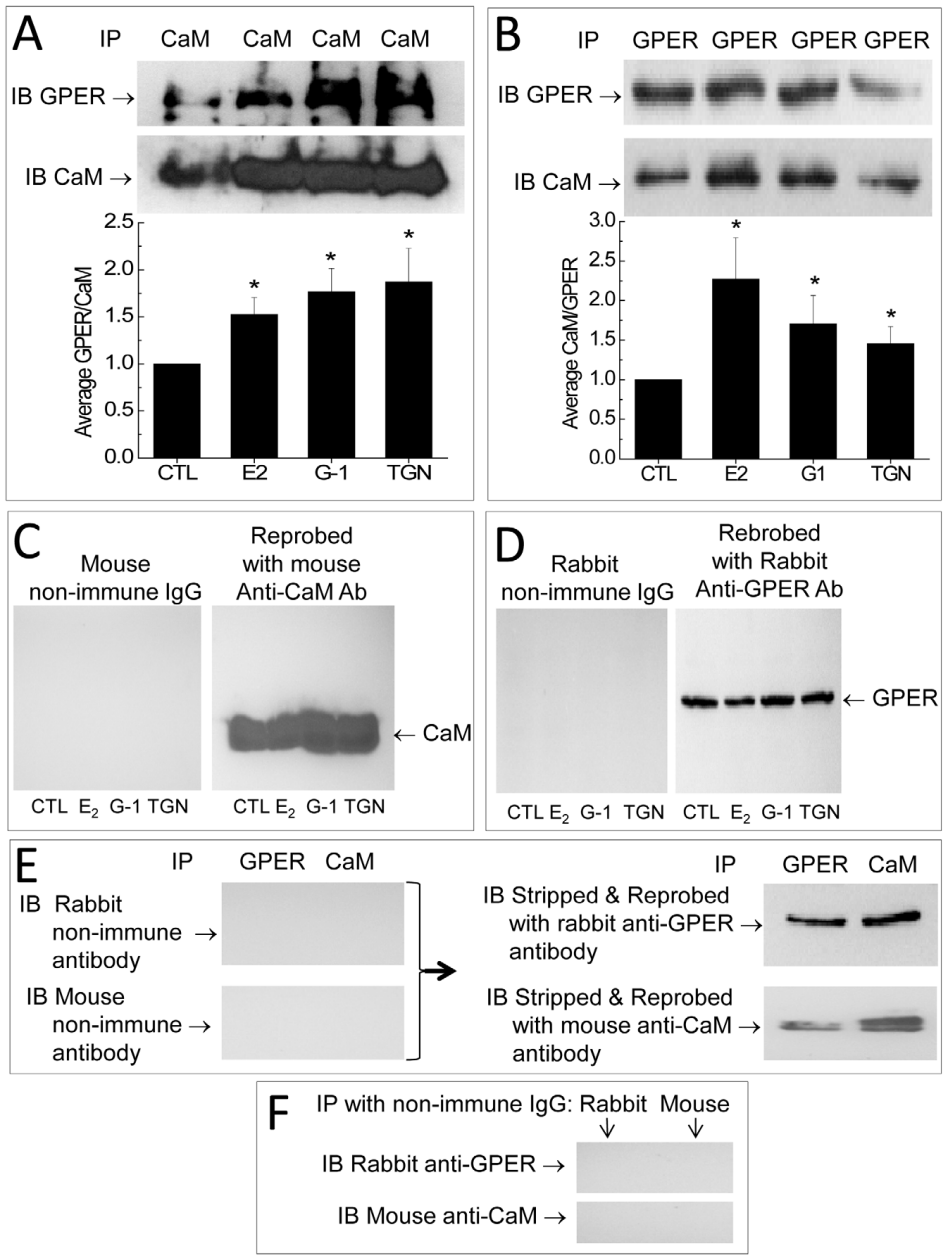
GPER forms a complex with CaM in primary VSMCs. Cells were treated acutely for 3 minutes with vehicle, 5β-estradiol (E_2_), 100 nM G-1 or 2 µM thapsigargin (TGN) in the presence of 1 mM extracellular Ca^2+^. (A) CaM was immunoprecipitated from the cell lysate; following electrophoresis of the pull down fractions, membrane was cut between the level of GPER and CaM and respective areas were probed for GPER and CaM using rabbit anti-GPER and mouse anti-CaM antibodies, respectively; (B) GPER was pulled down from the cell lysate, followed by probing for GPER and CaM as in (A). Histograms show average (n = 3) ratios of densitometric values of the prey protein to those of the bait proteins. (C) Total lysate from the co-IP reactions in (A) was first probed with mouse non-immune IgG (left panel). The membrane was subsequently stripped and reprobed with mouse-anti-CaM antibody (right panel). Note the absence (left) and presence (right) of the specific CaM bands in all samples. (D) Total lysate from the co-IP reactions in (B) was first probed with rabbit non-immune IgG (left panel). The membrane was then stripped and reprobed with rabbit anti-GPER antibody (right). Note the absence (left) and presence (right) of the specific GPER bands in all samples. (E) Immunoprecipitation was performed using rabbit anti-GPER antibody and mouse anti-CaM from lysate of cells treated with 5 nM E_2_ in the presence of 1 mM Ca^2+^. Following electrophoresis, membrane was cut between the level of GPER and CaM and probed separately with rabbit non-immune antibody (upper left) or mouse non-immune IgG (lower left). The same membranes were then stripped and reprobed with rabbit anti-GPER antibody (upper right) or mouse anti-CaM antibody (lower right). (F) Cell lysate from VSMCs treated as in (E) were immunoprecipitated with either rabbit (left lane) or mouse (right lane) non-immune IgG. Following electrophoresis, membrane was cut between GPER and CaM levels, and the membranes were probed with either rabbit anti-GPER antibody (upper) or mouse anti-CaM antibody (lower). *, p<0.05 from respective control values.

Estrogen activates many CaM-dependent processes. We considered the possibility that CaM might be involved in estrogen actions through GPER at the receptor level. We asked whether GPER interacts with CaM and whether this interaction requires GPER activation by specific ligand binding. To test this, primary VSMCs were acutely treated with vehicle or the endogenous ligand 17β-estradiol (E_2_, 5 nM), the GPER agonist G-1 (100 nM), or the SERCA pump inhibitor thapsigargin (2 µM) for 3 minutes in the presence of 1 mM extracellular Ca^2+^. Both E_2_ and G-1 have been demonstrated to trigger intracellular Ca^2+^ signals [13], [14], [44]–[48], and thapsigargin is a well-known stimulator of store-operated Ca^2+^ entry. GPER was immunoprecipitated from the lysate and the pull-down fractions were probed for CaM (Fig. 2A). Reciprocally, CaM was immunoprecipitated from the lysate and the pulled down fractions were subsequently probed for GPER (Fig. 2B). In both co-IP directions, CaM appears to associate with GPER under resting conditions. Both 17β-estradiol and GPER agonist G-1 apparently enhanced the association between GPER and CaM. Interestingly, thapsigargin, which stimulates Ca^2+^ entry independently of GPER activation, also produced a similar effect (Fig. 2A and 2B). To confirm specificity of the co-IPs, total cell lysate from a set of samples used in the co-IP reactions in Fig. 2A and 2B was first probed with non-immune IgGs from both mouse (Fig. 2C, left panel, species compatible with that of the anti-CaM antibody) and rabbit (Fig. 2D, left panel, species compatible with that of the anti-GPER antibody). These panels, predictably, showed no detectible bands. Following probing with the non-immune IgGs, the same membranes were stripped and re-probed with mouse anti-CaM antibody (Fig. 2C, right panel) or rabbit anti-GPER antibody (Fig. 2D, right panel). These panels now showed clear GPER and CaM bands, confirming the specificity of the antibodies used for GPER and CaM. To further confirm the specificity of the co-IPs in Fig. 2A and 2B, a control co-IP paradigm was tested in which GPER and CaM were immunoprecipitated from lysate of cells treated acutely with 5 nM E_2_, followed by immunoblotting with non-immune rabbit antibody (Fig. 2E, left upper panel) and mouse non-immune IgG (Fig. 2E. left lower panel). As predicted, these panels showed no detectible immunoprecipitates. The same membranes were then stripped and immunoblotted with rabbit anti-GPER antibody (Fig. 2E, right upper panel) and mouse anti-CaM antibody (Fig. 2E, right lower panel). Additionally, immunoprecipitation of lysate from E_2_-treated cells using the non-immune IgGs showed no detectible GPER or CaM upon immunoblotting with their specific antibodies (Fig. 2F, upper and lower panels, respectively).

### Identification of GPER’s CaM-binding Domains

**Figure 3:**
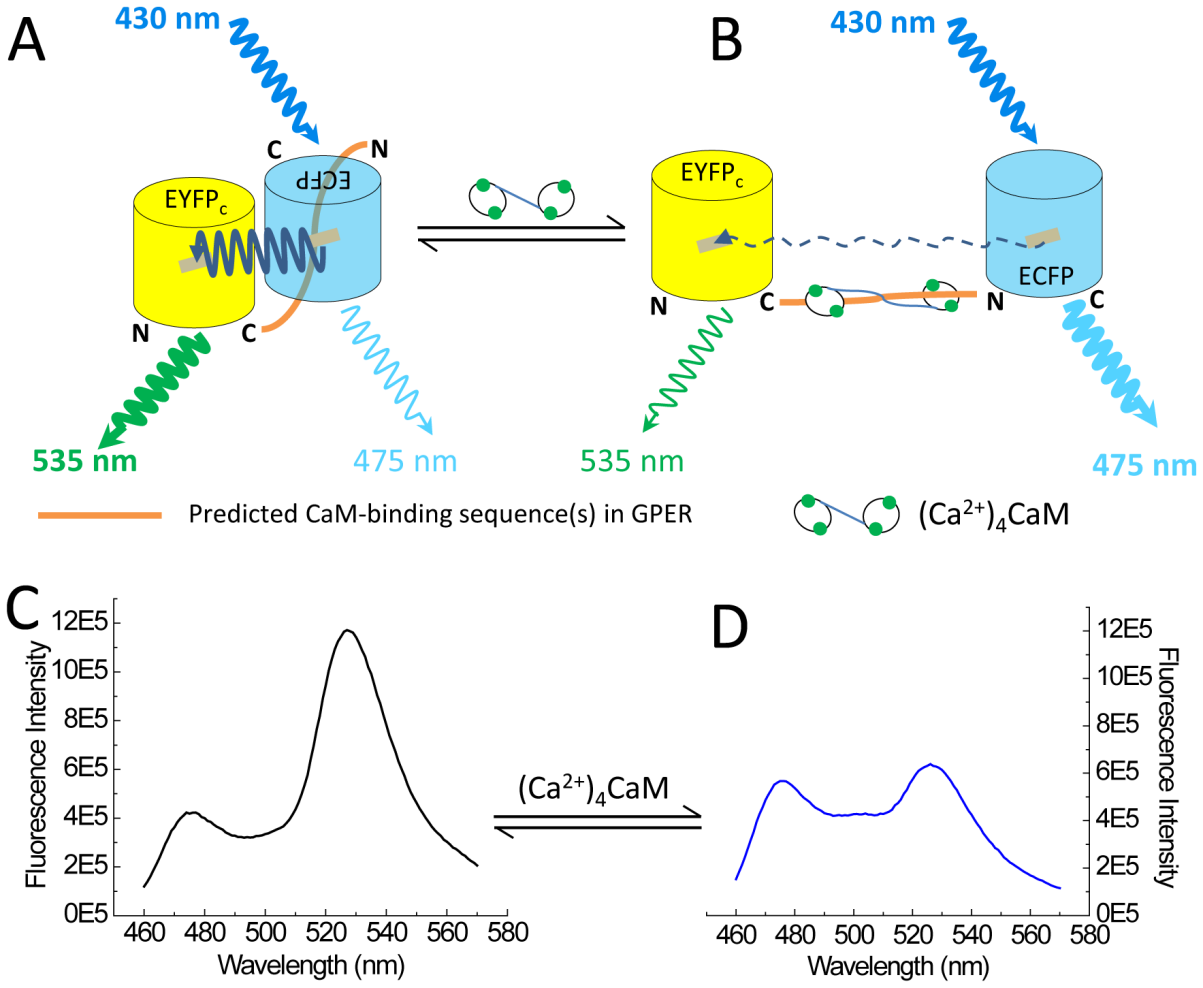
Design of biosensors to screen for CaM binding sequences in GPER. Biosensor is composed of a FRET donor ECFP and a FRET acceptor citrine EYFPc flanking each of the four submembrane domains in GPER and their truncated segments corresponding to our predicted CaM-binding sequences. The proximity between the donor and acceptor in the unbound state facilitate robust FRET when the donor ECFP is excited at 430 nm giving emission light at 475 nm, which in turn excites the acceptor EYFPc, which emits at 535 nm (*A*). Upon addition of CaM, if there is specific interaction with the GPER insert fragment, the conformational changes that occur upon specific interaction with CaM will disrupt FRET, decreasing emission from EYFPc while increasing that from donor ECFP. This signature spectral change identifies CaM binding (*B*). *C* and *D*, Typical spectrofluorometric response of biosensor in the absence of specific CaM binding to its linker (*C*), which corresponds to the biosensor configuration in (*A*), and in the presence of CaM and saturating concentration of Ca^2+^ (*D*), which corresponds to the biosensor configuration in (*B*).

The co-immunoprecipitation of GPER and CaM in VSMCs suggested that GPER might directly interact with CaM. In general, CaM-binding sequences do not belong to a common motif, so it is challenging to define CaM-binding domains in different proteins using sequence alignment [49]. For a GPCR, sub-membrane domains represent logical site(s) for these interactions. To confirm direct GPER-CaM interaction and locate the CaM-binding domains, we decided to use FRET technology as a new approach to confirm direct interaction and identify CaM-binding sites. The typical length of CaM-binding sequences, ∼20 amino acids, and the large conformational change produced upon CaM binding are ideal for FRET technology. FRET biosensors based on known CaM-binding sequences have been developed, modified and used successfully to monitor free Ca^2+^-CaM signals in cells [25], [26], [28], [43], [50]. However, FRET technique has not been used to locate unknown CaM-binding sequences. We believe that this technique could also be used to cost-effectively and precisely locate unknown CaM-binding domains in GPCRs and quantitatively determine their binding properties. Figure 3 recapitulates the principle of this approach. Given sufficient proximity of the energy donor ECFP and acceptor EYFPc, FRET is formed between these moieties upon excitation of ECFP at 430 nm (Fig. 3A). Specific binding of CaM to the linker causes a conformational change between the two fluorescent proteins and disrupts FRET, manifesting as an increase in the emission intensity of the donor (475 nm) and a corresponding decrease in that of the acceptor (535 nm, Fig. 3B). This signature behavior is specific for direct CaM-linker interaction. Ca^2+^ dependency of the interactions can be easily assessed by whether biosensor produces a response to CaM in the absence of Ca^2+^, or whether biosensor response in the presence of Ca^2+^ is abolished upon chelation of Ca^2+^ using a chelator such as BAPTA. Biosensor responses obtained in the presence of saturating Ca^2+^ level and incremental increases in purified CaM allow precise determination of apparent affinity of the interaction. Figures 3C and 3D show representative spectral changes that correspond to the scenarios depicted in Fig. 3A and Fig. 3B, respectively. We decided to use this approach to identify sequences in GPER that produce the lowest apparent K*_d_* values for their interactions with CaM. Another parameter of FRET-based biosensors is the dynamic range (DR) of their responses, operationally defined as the fold difference between the observed R*_min_* and R*_max_* (equation (1)). This value reflects the conformational change of the linker that occurs upon binding and was used as an additive factor in determining the precise location of the CaM-binding domains.

**Figure 4:**
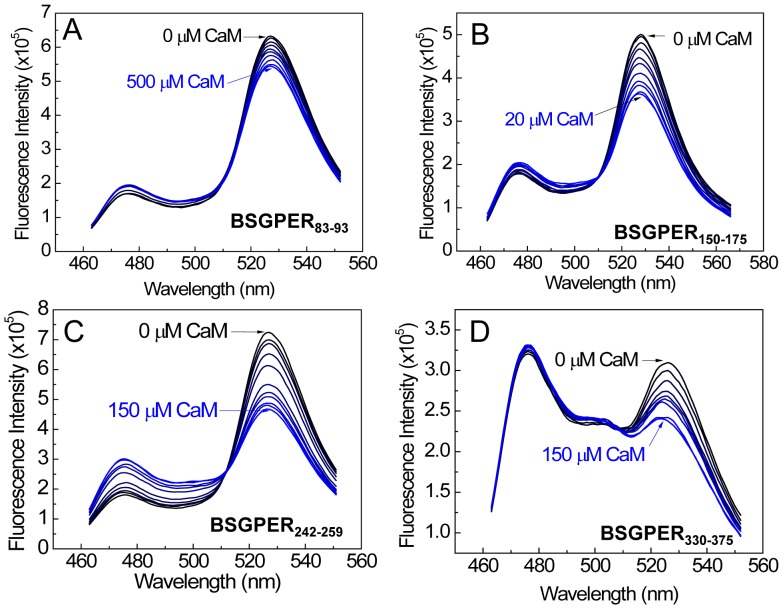
Initial screen of GPER’s submembrane domains for CaM binding. Incremental purified CaM was titrated as the entire emission spectra of BSGPER_83–93_ (SMD1, *A*) BSGPER_150–175_ (SMD2, *B*), BSGPER_242–259_ (SMD3, *C*) and BSGPER_330–375_ (SMD4, *D*) were monitored. Initial reaction mix contained 500 nM BSGPER_x_, 0.1 mg/mL BSA, and 3 mM Ca^2+^. Clear responses were observed for all SMDs.

**Figure 5:**
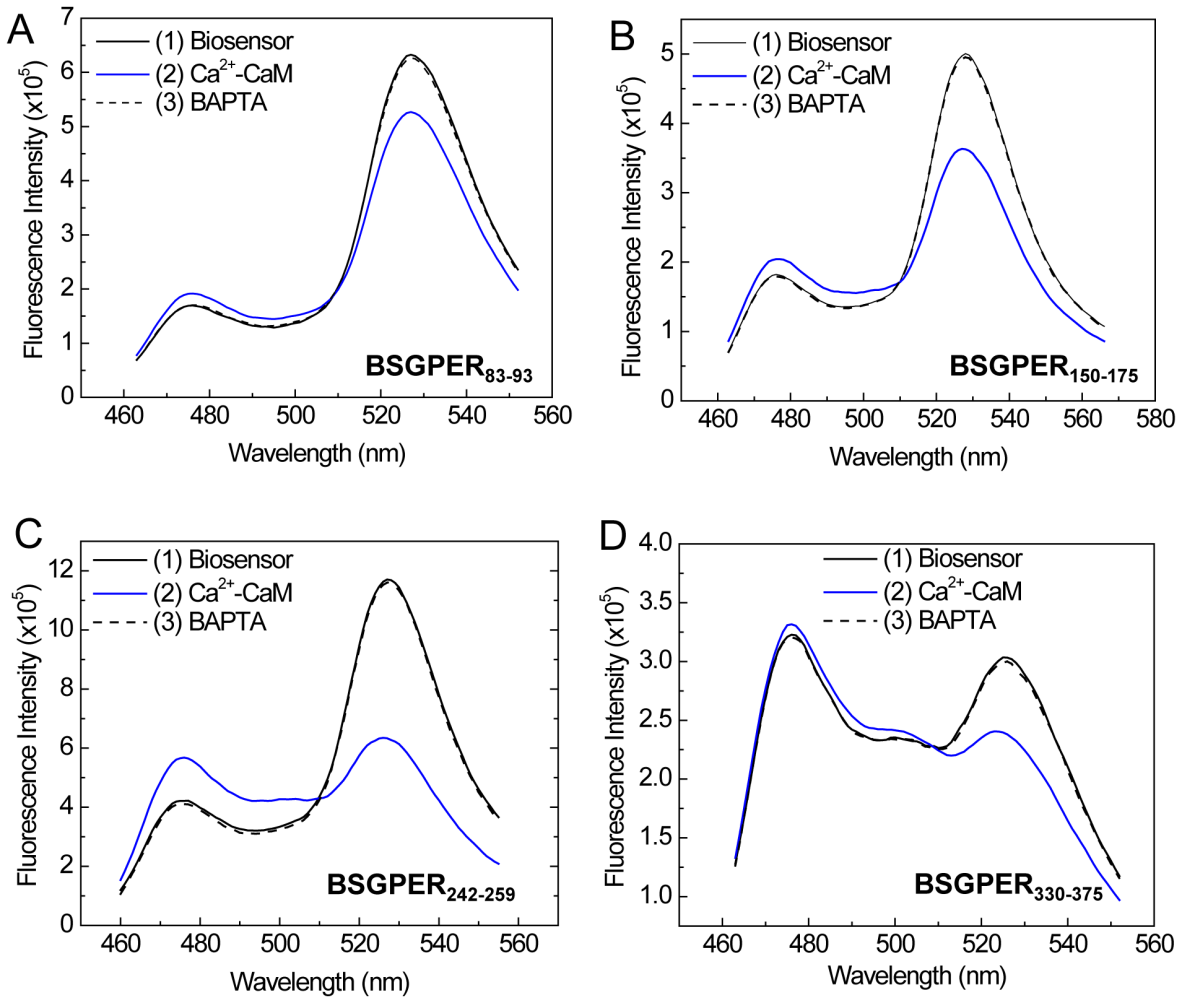
Reversibility and Ca^2+^ dependency of BSGPER_x_–CaM interactions. Saturating CaM concentrations identified in screening experiments (Figure 4) were added to a mixture of 500 nM BSGPER_83–93_ (A), BSGPER_150–175_ (B), BSGPER_242–259_ (C), and BSGPER_330–375_ (D) in the presence of 0.25 mM Ca^2+^ (3 mM Ca^2+^ for the case of BSGPER_83–93_) (solid blue traces), followed by addition to 2 mM of BAPTA (5 mM for the case of BSGPER_83–93_) to chelate Ca^2+^ (dotted black traces). Note the reversal of the responses to very close to the level before the addition of Ca^2+^-CaM (black solid traces), indicating Ca^2+^-dependent interactions between SMD1-4 with CaM.

As an initial screen, we inserted the sub-membrane domains (SMDs) of GPER as linkers between the FRET donor ECFP and acceptor EYFPc and screened for direct interactions with purified CaM. Fig. 4 shows the emission spectral changes of these biosensors upon incremental additions of Ca^2+^-saturated CaM (Ca^2+^-CaM). BSGPER_83–93_, whose linker corresponds to GPER’s SMD1, showed reciprocal responses in its emission at λ475 nm and λ535 nm only to fairly high CaM concentrations (Fig. 4A), and was saturated at 500 µM. BSGPER_150–175_, BSGPER_242–259_ and BSGPER_330–375_, whose linker sequences correspond to GPER’s SMDs 2–4, also display reciprocal changes in its emission intensities at λ475 nm and λ535 nm upon additions of Ca^2+^-CaM (Fig. 4B–D), albeit with different dynamic ranges (Table 1). The lower dynamic range of BSGPER_330–375_ (Fig. 4D) is likely due to its longer linker sequence. To confirm Ca^2+^-dependency of these interactions, saturating concentrations of CaM determined from titrations similar to those shown in Fig. 4 were added to a mixture of 500 nM BSGPER_x_ and 250 µM Ca^2+^ (3 mM Ca^2+^ for BSGPER_83–93_). After maximal responses were obtained for BSGPER_83–93,_ BSGPER_150–175,_ BSGPER_242–259,_ BSGPER_330–375_ (solid blue traces, Fig. 5A–D), 2 mM BAPTA (5 mM for BSGPER_83–93_) was added to chelate Ca^2+^ in the mixture. This addition completely reversed the responses for these biosensors (dotted traces, Fig. 5A–D). These data indicate direct Ca^2+^-dependent interaction between CaM and GPER’s submembrane domains 1–4.

**Table 1:** BSGPER_x_, dynamic ranges and affinities for CaM.*

BSGPER_X_	SMD	Dynamic Range	Apparent K_d_ (µM)
BSGPER_83–93_	1	1.31±0.01¶	136.62±6.56
BSGPER_150–175_	2	1.61±0.03	0.44±0.03
BSGPER_150–170_	2	1.34±0.02¶	3.41±0.09
BSGPER_242–259_	3	2.65±0.05	8.01±0.49
BSGPER_330–351_	4	2.50±0.03	1.40±0.16
BSGPER_330–345_	4	2.45±0.04	1.60±0.22

Data are means ± SD of at least three independent determinations.

*All K*_d_* values are significantly different (p<0.05) from one another.

¶, these values are different (p<0.05) from the remaining dynamic range values.

**Figure 6:**
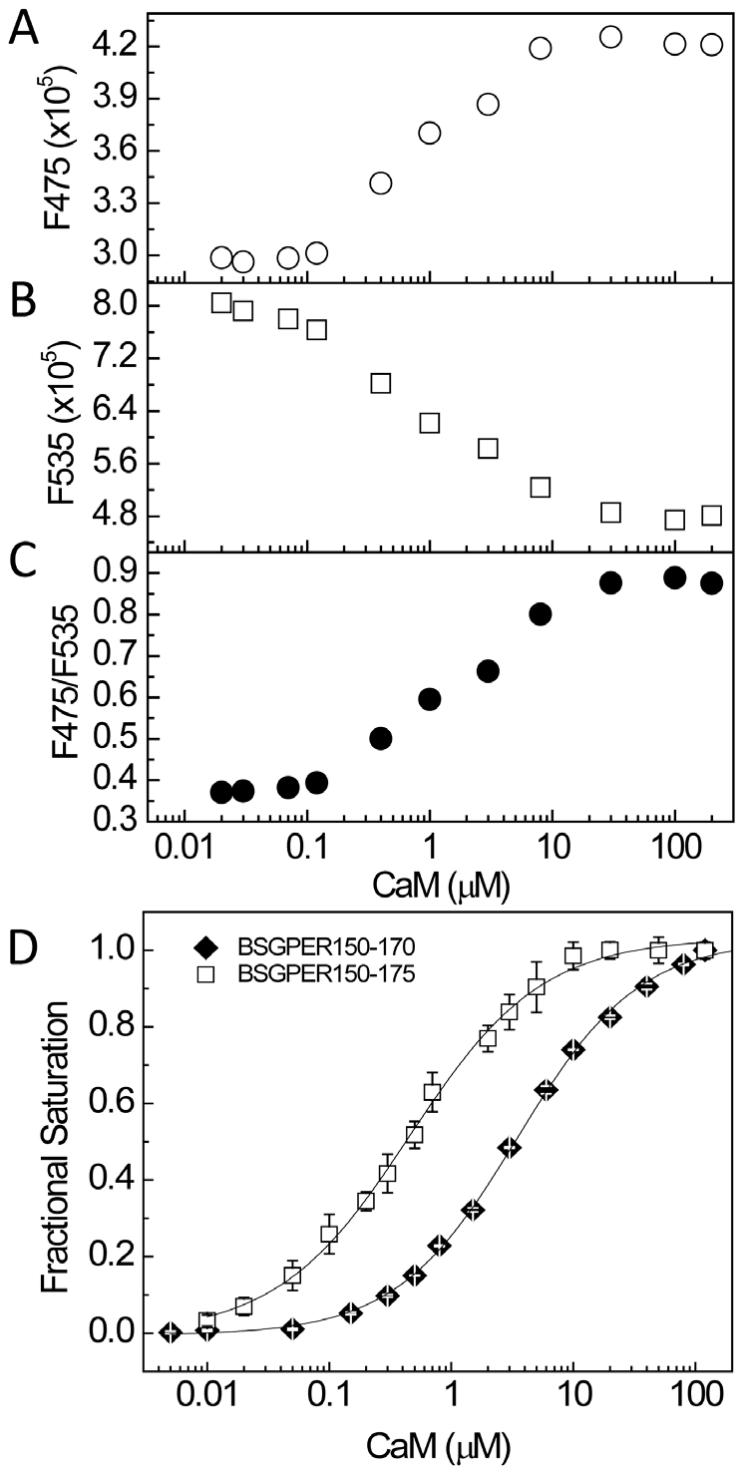
Identification of the correct CaM-binding sequence in SMD2. A–C. Typical experiment to determine K*_d_* value and dynamic range of BSGPER_x_. Incremental Ca^2+^-saturated CaM was titrated as in Fig. 4 into a mixture of BSGPER_150–170_ and titration buffer, except that only the fluorescence emission intensities at 475 nm (*A*) and 535 nm (*B*) and their ratios (*C*) were collected for 10 s following each addition. Dynamic range and fractional response were calculated and K*_d_* value derived as described under Experimental Procedure. *D*, Difference in the affinity of BSGPER_150–170_ (closed diamonds) and BSGPER_150–175_ (open squares) revealed the role of the basic patch (a.a. 170–175) in determining the affinity for CaM binding to SMD2 and confirmed the entire SMD2 (a.a. 150–175) is involved in CaM binding.

To narrow down the precise CaM-binding domains in GPER, our strategy was to adjust the length of the linker sequences in keeping if possible with common CaM-binding motifs [51] and then screen for biosensors that yield the lowest K*_d_* values for CaM and largest dynamic ranges. BSGPER_83–93_ (SMD1) represents a fairy short sequence (11 residues) that does not follow any known CaM-binding motifs and yet apparently binds CaM in a strictly Ca^2+^-dependent fashion, albeit with relatively low affinity. BSGPER_150–175_ (SMD2) contains a 20-a.a. segment (a.a. 150–170) of hydrophobic residues interspersed among positively charged residues, while the remaining part (a.a. 170–175) is a patch of dense basic, positively charged residues (Fig. 1B). Although the positions of bulky hydrophobic residues in the 150–170 segment do not fit any common CaM-binding motifs [51], the composition of hydrophobic and positively charged residues does suggest potential of a CaM-binding domain. BSGPER_150–170_ was thus constructed for comparison with BSGPER_150–175_. This comparison would also reveal the role of the basic patch (a.a. 170–175) in CaM binding to SMD2. Figure 6A–C shows a typical experiment to determine the apparent K*_d_* value for CaM-BSGPER_x_ interaction using the example of BSGPER_150–170_. Incremental purified CaM was added to a mixture of biosensor and saturating Ca^2+^ concentration, and the fluorescence emission intensities of donor ECFP (Fig. 6A, F475), acceptor EYFPc (Fig. 6B, F535) and a ratio between these values (Fig. 6C) were simultaneously obtained. Clear reciprocal changes in F475 and F535 intensities were observed as Ca^2+^-CaM was added, which corresponds to changes in the ratio between them. Fractional responses of the biosensor was calculated plotted as a function of Ca^2+^-CaM, and the apparent K*_d_* values was obtained as described under Experimental Procedures. Interestingly, BSGPER_150–170_ demonstrated a ∼8-fold weaker affinity than BSGPER_150–175_ (Fig. 6D and Table 1). This result clearly indicates that the entire SMD2 participates in CaM interaction. It also demonstrates the importance of the patch of basic, positively charged residues (170–175) in CaM binding to SMD2.

**Figure 7:**
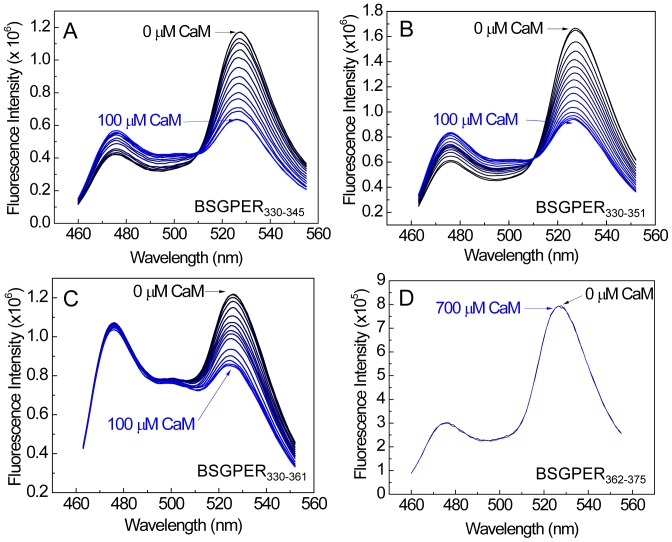
Narrowing down the precise CaM-binding domain in SMD4 using different biosensors. Ca^2+^-saturated CaM was titrated as the entire emission spectra of BSGPER_330–345_ (*A*), BSGPER_330–351_ (*B*), BSGPER_330–361_ (*C*) and BSGPER_362–375_ (*D*) were monitored. Experiments were performed as described in Figure 6.

**Figure 8:**
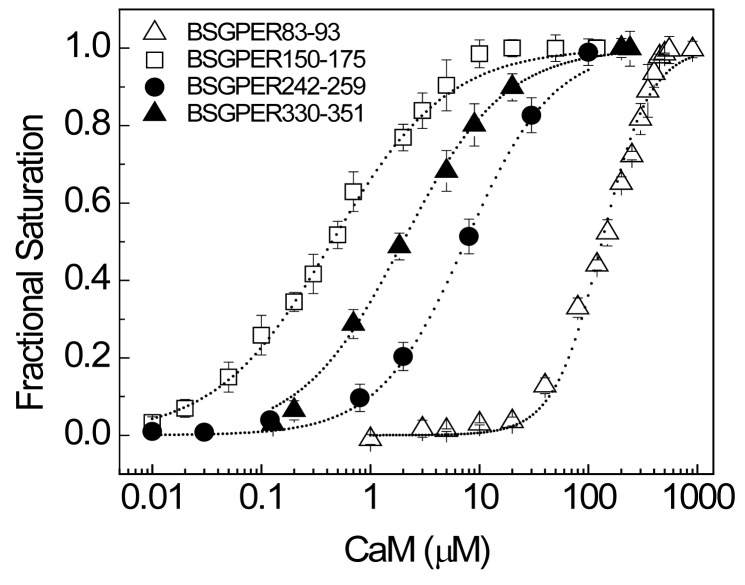
Distinct affinities for Ca^2+^-CaM of the three CaM binding domains in GPER. Average fractional responses obtained from titrations of Ca^2+^-CaM to BSGPER_83–83_ (open triangles), BSGPER_150–175_ (open squares), BSGPER_242–259_ (closed circles) and BSGPER_330–351_ (closed triangles) were plotted as a function of CaM.

The 18-residue SMD3 (a.a. 242–259) contains several hydrophobic residues interspersed among a very dense group of basic residues. The hydrophobic residues here again do not appear to follow any of the common motifs of CaM-binding sequences. Given the short length of this submembrane domain, it is likely that the entire domain interacts with CaM (Fig. 4C). As for SMD4, we noted that BSGPER_330–375_ showed limited dynamic range, due probably to its long linker sequence (Fig. 4D). The SMD4 sequence shows in its juxtamembrane region a potential CaM-binding domain. Additional BSGPER_x_ were thus constructed with linkers corresponding to fragments a.a. 330–345, 330–351, and 330–361. To rule out a role of the C-terminal end of SMD_4_ in its interaction with CaM, a biosensor was also generated based on a.a. 362–375. As shown in Fig. 7A–C, BSGPER_330–345_, BSGPER_330–351_ and BSGPER_330–361_ all display robust responses to Ca^2+^-CaM, in agreement with the prediction that the CaM-binding domain in SMD4 is located in its juxtamembrane segment. Consistently, BSGPER_362–375_ does not respond to Ca^2+^-CaM, even at such high concentration as 700 µM CaM (Fig. 7D); this serves as a negative control that CaM does not affect ECFP and EYFP fluorescence. BSGPER_330–351_ displays slightly larger dynamic range and higher affinity than BSGPER_330–345_, while BSGPER_330–361_ shows a limited dynamic range. These data indicate that a.a. 330–351 represents the CaM-binding sequence in SMD4. The data presented so far clearly indicate that GPER possess four distinct CaM-binding sites in all of its submembrane domains with disparate affinities. An aggregate plot representing these remarkable differences is shown in Fig. 8. The apparent K*_d_* values for these interaction are significantly different from one another (p<0.05) and are summarized in Table 1 alongside their dynamic ranges.

### Ca^2+^ Sensitivities of the Interactions between CaM and GPER’s CaM-binding Domains

**Figure 9:**
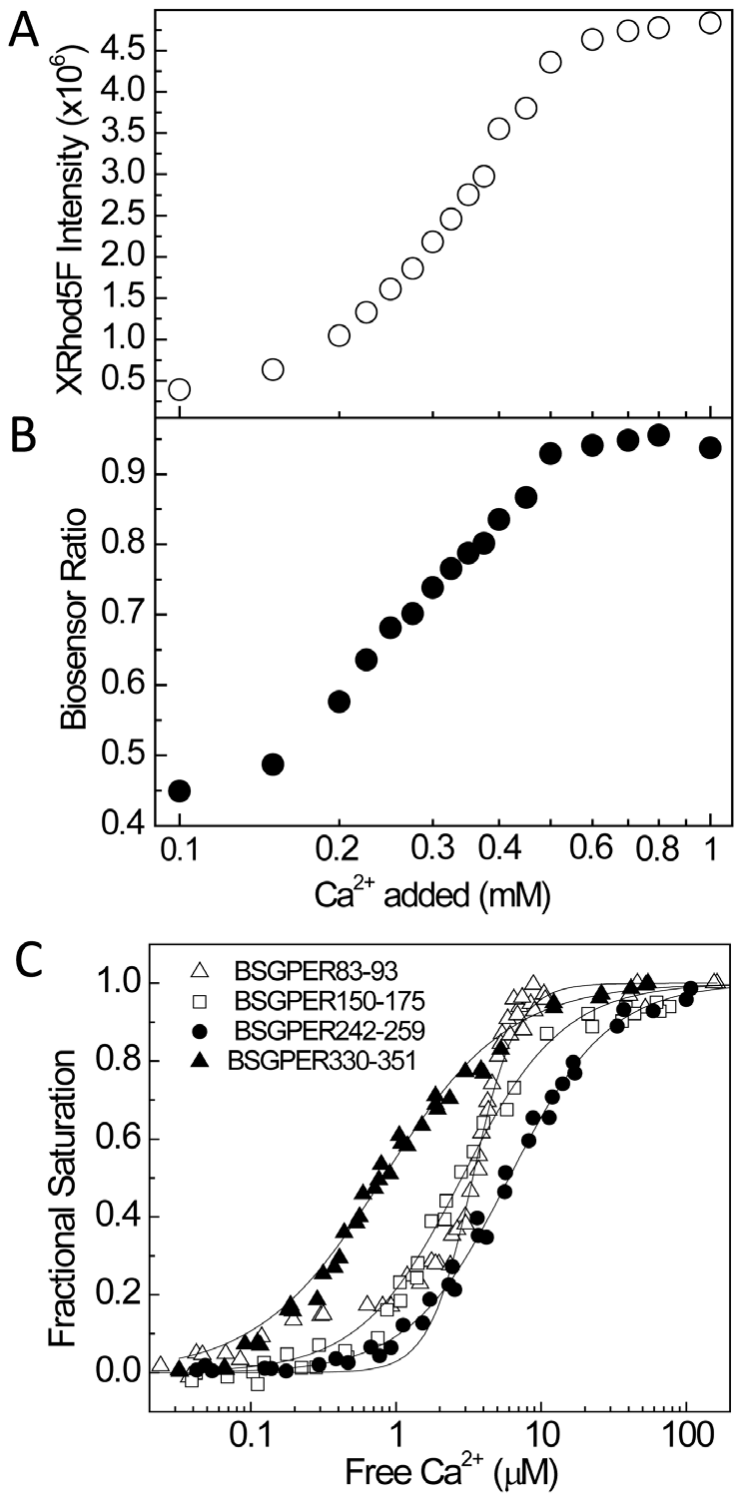
Determination of the Ca^2+^ sensitivities of the interactions between BSGPER_x_ and CaM. (A & B) Typical experiment to determine EC50(Ca^2+^) value for BSGPER_x_-CaM interactions. Reactions contained 0.5 µM BSGPER_x_, 2 µM XRhod-5F, 0.25 mM BAPTA, and saturating CaM concentration (obtained from K*_d_* titrations). Incremental aliquots of Ca^2+^ were added as the emission intensities of XRhod-5F (*A*, open circles) and biosensor ratios (*B*, closed circles) were simultaneously monitored. Fractional biosensor response were plotted against free Ca^2+^ calculated from XRhod-5F response as described under Experimental Procedure. *C*, Titrations showing Ca^2+^ sensitivity of the interactions between CaM and BSGPER_83–93_ (open triangles), BSGPER_150–175_ (open squares), BSGPER_242–259_ (closed circles), BSGPER_330–351_ (closed triangles). Fits were performed on aggregate data (shown) of at least three independent experiments. Starting reaction mix for BSGPER_83–93_ titrations contained 3 mM BAPTA instead of 0.25 mM BAPTA.

The disparate affinities for CaM binding of the four domains identified above suggest that they may interact with CaM under different physiological scenarios. Given the complete Ca^2+^-dependent nature of these interactions (Fig. 5), knowledge of their Ca^2+^ sensitivities would be very helpful in determining the roles of CaM binding to each submembrane domain when studying GPER’s physiological functions. The best approach was to simultaneously monitor BSGPER_x_ fractional responses and free Ca^2+^ concentrations in the presence of saturating CaM and increasing amounts of free Ca^2+^ measured using an appropriate Ca^2+^ indicator. Based on the K*_d_* values determined (Table 1), saturating CaM concentrations for the biosensors were known. The choice of a suitable Ca^2+^ indicator to do this depends on two factors. First, its excitation and emission spectra should not interfere with those of the biosensors. Second, it must have an apparent K*_d_* for Ca^2+^ that is close enough to the Ca^2+^ sensitivity of the biosensor being tested, which could be quickly assessed by simultaneous measurement of both biosensor and the Ca^2+^ indicator responses as Ca^2+^ is titrated to the mixture. A wide range of commercially available Ca^2+^ indicators made this task relatively easy. A few Ca^2+^ indicators were tested, including indo-1, Mag-indo1, and XRhod-5F. XRhod-5F represented an ideal Ca^2+^ indicator for use with these biosensors, due to the closeness of its K*_d_* values to the estimated Ca^2+^ sensitivities of the biosensors and the fact that both excitation (λ580 nm) and emission (λ605 nm) peaks are far to the right to the emission peak of the EYFPc moiety of the biosensors. Figure 9A and B show changes in XRhod-5F (A) and biosensor ratio (B) simultaneously monitored as Ca^2+^ was titrated to a mixture of biosensor, saturating concentration of CaM. Starting mixture contained 0.25–3 mM BAPTA to guarantee nominally Ca^2+^-free condition at the beginning of the titration.

Figure 9C shows plots of the fractional responses of BSGPER_83–93,_ BSGPER_150–175_, BSGPER_242–259_, and BSGPER_330–351_ to saturating CaM concentrations as a function of free Ca^2+^. The four CaM-binding domains display significantly different Ca^2+^ sensitivities in their interaction with CaM. BSGPER_150–175_, which has the strongest affinity among the four (Fig. 8), only displays an intermediate Ca^2+^ sensitivity compared to the others (Fig. 9C).

**Figure 10:**
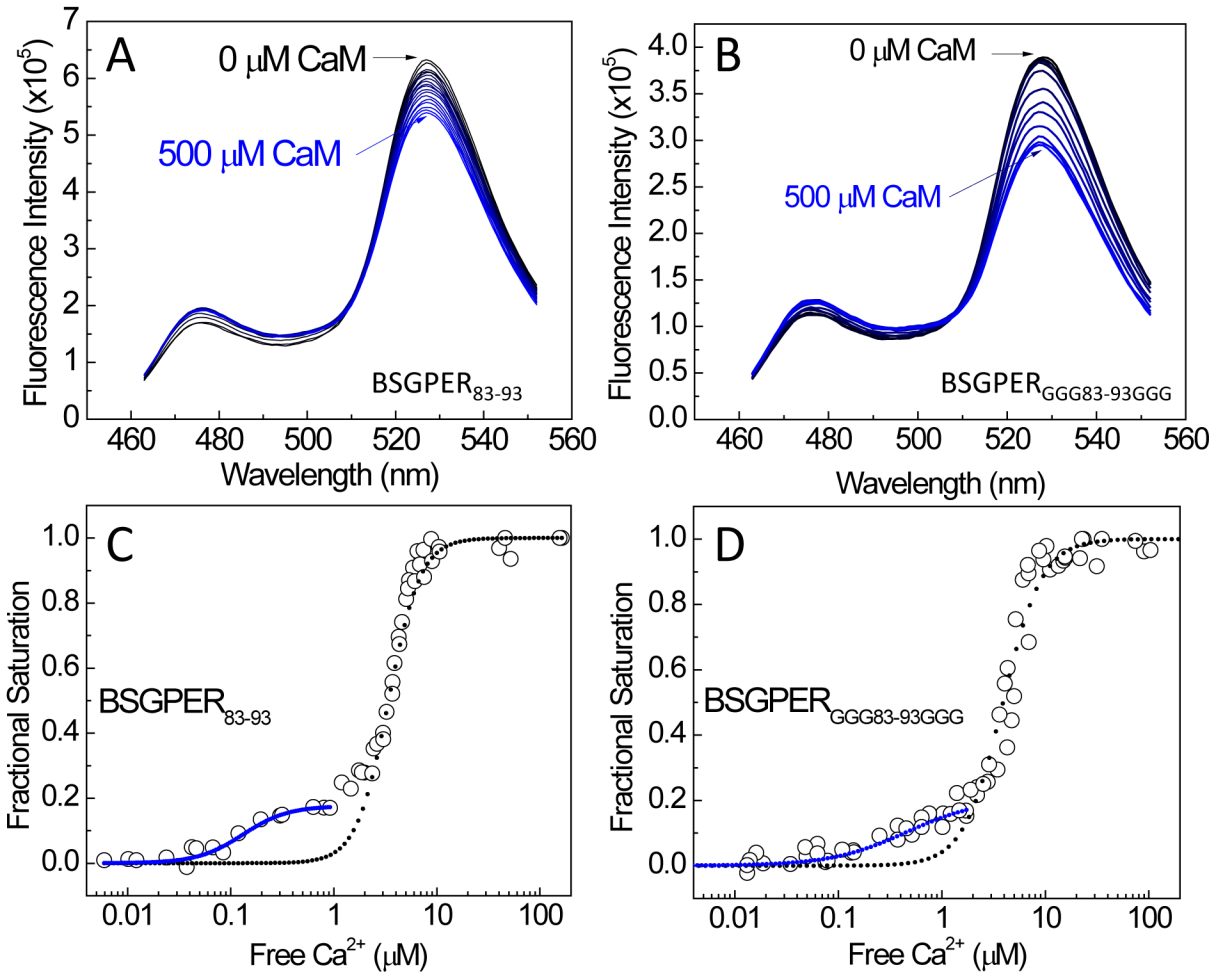
CaM titrations and Ca^2+^ titrations for BSGPER_83–93_ and BSGPER_GGG83–93GGG._ (A & B) Typical CaM titrations for BSGPER_83–93_ (A) and BSGPER_GGG-83–93GGG_ (B). Starting reaction mix contained 0.5 µM biosensor, 3 mM Ca^2+^, 0.1 mg/mL BSA. (C & D) Aggregate data from Ca^2+^ titrations for the interactions between CaM and BSGPER_83–93_ (C) and BSGPER_GGG83–93GGG_ (D). Starting reactions contained 0.5 µM biosensor, 700 µM CaM, 3 mM BAPTA, 0.5 mM BSA, 2 µM XRhod-5F. Fits for the entire set of data were shown in black dotted lines. Fits for the separate first phase of data (up to measured free Ca^2+^ values of 1 µM) were shown in blue lines.

Interestingly, BSGPER_83–93,_ which binds CaM with the lowest affinity among the four domains identified (Fig. 8 and Table 1), displays a clearly biphasic Ca^2+^ response curve (Fig. 9C), the majority of which with a much higher Hill coefficient. To rule out a potential complication due to the fairly short distance between the FRET donor and acceptor of this biosensor (11 residues), which might limit access of CaM to the linker, we decided to lengthen the linker by adding three glycine residues to each end of SMD1 in this biosensor configuration, generating BSGPER_GGG83–93GGG_. BSGPER_GGG83–93GGG_ binds CaM with essentially identical K*_d_* value with BSGPER_83–93_ (not shown), despite a slightly increased dynamic range due to a longer linker (Fig. 10B vs 10A). Interestingly, simultaneous determinations of BSGPER_GGG83–93GGG_ response and free Ca^2+^ values still demonstrated the biphasic behavior (Fig. 10D) identical to that of BSGPER_83–93_ (Fig. 10C). These results ruled out the short distance between the FRET pair as an obstacle for CaM access and suggested that the biphasic nature of the Ca^2+^ titration curves is due perhaps to the composition of the SMD1 sequence. Examinations of the response curves in Fig. 10C and 10D show two distinct species of SMD1-CaM complexes with respect to their Ca^2+^ sensitivity. BSGPER_83–93_–CaM species 1 are complexes formed at a low free Ca^2+^ concentration (below 1 µM free Ca^2+^). Fits of this species formation against free Ca^2+^ (blue curve, Fig. 10C) yielded an average EC50(Ca^2+^) value of 0.13±0.02 µM. Analyses of BSGPER_GGG83–93GGG_–CaM species 1 (blue curve, Fig. 10D) responses yielded essentially identical value. It is interesting to note that these EC50(Ca^2+^) values are well in the range of resting free cytoplasmic Ca^2+^ concentrations in cells. BSGPER_83–93_–CaM species 2 are complexes formed at drastically higher free Ca^2+^ concentrations than species 1. Fits of the entire population formed as a function of free Ca^2+^ values follow the behavior of this more abundant species (black dotted lines, Fig. 10C), which yielded an average EC50(Ca^2+^) value of 3.71±0.13 µM. This value is well within cytoplasmic Ca^2+^ values measured within the sustained phase of vasopressin-induced Ca^2+^ entry in smooth muscle cells [52]. Fits of species 2 formation also yielded significantly higher Hill coefficients than those of species 1. Table 2 summarizes the different EC_50_(Ca^2+^) values and the Hill coefficients for the four CaM binding domains in their Ca^2+^-dependent interactions with CaM.

**Table 2:** EC_50_(Ca^2+^) values and Hill coefficients for interactions between CaM and GPER’s submembrane domains.*

Complexes	SMD	EC50(Ca^2+^)(µM)	Hill Coefficient
CaM-GPER_83–93_ (Species 1)	1	0.13±0.02	1.99±0.14*
CaM-GPER_83–93_ (Species 2)	1	3.71±0.13	2.53±0.15* ¶
CaM-GPER_150–175_	2	2.38±0.13	1.21±0.09
CaM-GPER_242–259_	3	5.15±0.55	1.43±0.11
CaM-GPER_330–351_	4	0.75±0.05	1.18±0.08

Data are means ± SD of at least 3 independent determinations.

*All EC50(Ca^2+^) values are significantly different (p<0.05) from one another; Hill coefficients for GPER83–93sp1 and GPER83–93sp2 are different (p<0.05) from the rest.

¶, significantly different (p<0.05) from GPER83–93 (Species 1).

## Discussion

Identification of CaM-binding domains in CaM-dependent proteins has traditionally involved synthesis of peptides that correspond to the predicted binding sequence and study of CaM-peptide interaction or of the interactions between CaM and the wild-type protein or a mutant with deletion of the predicted CaM binding sequence. For a G protein-coupled receptor, purification of the entire protein remains a challenge. The lack of an exhaustive approach to identify all CaM-binding domains in a GPCR and determine the Ca^2+^ sensitivity of their interactions with CaM has made it difficult to fully assess the potential roles of CaM in GPCR biology.

FRET biosensors have been developed based on known CaM-binding sequences to measure free Ca^2+^-CaM levels in cells [25], [26], [28], [50], [53]. In our opinion, FRET technology offers a simple alternative approach to identify unknown CaM-binding sequences in GPCRs that provides several advantages. First, typical CaM-binding domains are of ∼20 a.a. in length and represent ideal linkers for outstanding FRET efficiency. Using apparent K*_d_* values and dynamic range of biosensors, a parameter introduced in this paper, the length of linker based on a predicted domain can be easily adjusted until the accurate domain responsible for CaM binding is identified. For GPCRs, the submembrane domains are short fragments that allow for easy screening using this approach. Second, biosensors can be expressed and purified with relative ease and cost effectiveness. This is particularly helpful when there are multiple CaM-binding sites in the same protein, as in the case of GPER. Third, since FRET is proportionate to the inverse 6^th^ power of the molecular distance between the donor and acceptor fluorophores, the technique is very sensitive and therefore allows highly quantitative characterization of the interaction between CaM and its binding domains. Fourth, specific CaM binding to the linker sequence only involves changes in the fluorescence intensities of the donor and acceptor fluorophores and not any shifts in their spectral peaks, facilitating precise quantitation. Finally, the Ca^2+^ sensitivity of CaM-target interactions can be measured with high precision using the approach described in this paper. Knowledge of this sensitivity allows prediction of the physiological scenarios that facilitate interactions between CaM and the individual sub-membrane domains of a GPCR. This is particularly true given the constantly trafficking nature of GPCRs through different cellular locales, where the free Ca^2+^ concentrations may vary substantially.

In this study, we have utilized FRET biosensor technique for the first time to demonstrate direct interaction between GPER and CaM and identify four distinct CaM-binding sequences in the submembrane domains of this receptor. The majority of CaM binding domains identified so far in GPCRs are located in either the third submembrane domain or the juxtamembrane section of the fourth. To our knowledge, GPER is the first GPCR identified to possess four CaM-binding domains. Detailed characterizations of biosensor responses indicate that these domains bind Ca^2+^-CaM with significantly different affinities in the sub- to high micromolar ranges. The sensitive nature of the biosensor technique has enabled identification of the role of different fragments in the sequence under study in the interaction with CaM. For example, fragment 150–170 in GPER’s SMD2 binds CaM, but with an apparent affinity 8-fold lower than that of fragment 150–175. This clearly indicates an important role of the basic patch 170–175 in CaM binding to SMD2. The apparent K*_d_* values are obtained in vitro on isolated purified biosensors and, like values obtained using synthetic peptides, are not necessarily the same as in the entire receptor, whose expression and purification remains a challenge. Nevertheless, they provide a useful guide to predict interactions that might occur where GPER resides in its cycle in the cell. With the order in affinity of SMD2> SMD4> SMD3> SMD1, one would predict that SMD2 would be the first to bind CaM in response to a Ca^2+^ signal produced at the receptor’s location in cells. In addition, the identification of these domains provides a basis for future investigations of the roles of CaM in the various interactions at these locations between GPER and other proteins in cells.

As all of these interactions are Ca^2+^-dependent, knowledge of the Ca^2+^ sensitivity of each domain’s interaction with CaM becomes helpful in predicting the physiological scenarios in which each might occur. The concurrent determination of free Ca^2+^ concentrations and CaM-SMD interactions using responses of a suitable Ca^2+^ indicator and FRET biosensor allows highly precise determination of the Ca^2+^ sensitivities of these interactions. We believe that this approach avoids some potential difficulties associated with using Ca^2+^ buffers. One such would be uncertainty in the amount of free Ca^2+^ contained in biosensor stocks or CaM stocks, which would require complete Ca^2+^ chelation of these stocks prior to mixing with buffers, or uncertainly of the effect of dilution from additions of stock solutions. Based on the EC_50_(Ca^2+^) values determined, SMD2, while apparently being the most avid binder of CaM among the four SMDs, only possesses an intermediate Ca^2+^ sensitivity (EC_50_(Ca^2+^) ∼2.3 µM) for its interaction with CaM. Knowledge of the free Ca^2+^ concentrations produced in different physiological scenarios then can be used together with these values in predicting what interactions will take place in cells. Indeed, from our co-immunoprecipitation data, it is obvious that GPER and CaM are in the same complexes even at resting conditions. Based on the condition of the co-IP reaction, this would at first sight seem to be a Ca^2+^-independent interaction; however, based on the Ca^2+^ sensitivity data presented in Fig. 10 and Table 2, it is apparent that SMD1-CaM complexes can be formed at very low free Ca^2+^ concentration (species 1), with an EC50(Ca^2+^) value of ∼ 130 nM, a value in the range of resting cytoplasmic Ca^2+^ concentrations in many cell types. So from the standpoint of free Ca^2+^ value, this interaction can certainly occur in unstimulated cells. From the standpoint of available CaM, it is noteworthy that the interaction between CaM and SMD1 is of low affinity. So the question arises as to whether this interaction can occur in cells. It has been demonstrated that CaM is a limiting factor in cells and CaM targets compete for Ca^2+^-dependent interactions with CaM [25], [26], [28], [54]. However, at resting Ca^2+^ levels, the competition among targets for Ca^2+^-dependent interaction with CaM must be much reduced, as only those interactions that are highly sensitive to Ca^2+^, such as that of SMD1-CaM species 1, can take place. A similar case was recently demonstrated, where the interaction between endothelial nitric oxide synthase and CaM, and hence nitric oxide production, can occur at fairly low free Ca^2+^ concentrations (EC50(Ca^2+^) ∼ 150 nM) and that phosphorylation of the synthase substantially reduces, albeit by no means negate, the Ca^2+^ dependency of the interactions (EC50(Ca^2+^) ∼ 50 nM) [55], [56]. These observations have contributed to the explanation of the effects of eNOS phosphorylation or shear stress to increase basal nitric oxide production that previously was thought to be Ca^2+^-independent. In the case of GPER_83–93_-CaM complex species 1, the biochemical results obtained here lend a nice plausible interpretation of the co-IP data obtained in control cells. In addition, it is noteworthy that GPER has been found in a number of different locations in cells in addition to the plasma membrane [57], including the ER [58] and the trans-Golgi-proteasome network [59]. Under resting conditions, Ca^2+^ values in the Golgi network have been shown to be in the range of 100–250 µM, while that in the ER can reach above 450 µM [60], [61]. Given these values and the potential locations of GPER, it is not out of the question that interactions between CaM and the other SMDs of GPER can also occur in unstimulated cells, since the EC50(Ca^2+^) values for these interactions are all in the sub- or low micromolar range (Table 2). Of course, we cannot rule out an alternative possibility that other interaction partner(s) with GPER rather than CaM may also bind CaM with very high Ca^2+^ sensitivity or in a Ca^2+^-independent fashion, and showed up in the GPER pull-down fractions probed for CaM and vice-versa, under resting conditions. Nevertheless, our in-vitro data clearly demonstrate absolute Ca^2+^ dependence of the interactions between CaM and GPER and provide parameters that can sufficiently explain the co-IP data under resting condition.

Under stimulated conditions, it has been shown that vasopressin-induced SR Ca^2+^ release from VSMCs can raise cytoplasmic Ca^2+^ level above 1 µM, while sub-plasma membrane Ca^2+^ can reach 45 µM and remain above 5 µM for a sustained period during subsequent Ca^2+^ entry [52]. Data from Fig. 9C, 10C–D and Table 2 then allow prediction that in addition to SMD1-CaM species 1, SMD4-CaM complexes can easily form in the cytoplasm in response to SR release Ca^2+^ signal, while SMD3 and 2 can interact with Ca^2+^-CaM during the peak and sustained phase of agonist-induced Ca^2+^ entry in VSMCs. In this regard, it is interesting to consider the observation that thapsigargin, which stimulates store-operated Ca^2+^ entry independently of estrogen activity, enhanced the association between CaM and GPER independently of specific ligand binding in VSMCs (Fig. 1). An interesting possibility to investigate from this result is whether a generic cytoplasmic Ca^2+^ signal can activate GPER or alter its functions as a G protein-coupled receptor by promoting its multi-level interactions with CaM. Consistent with this idea, it has been demonstrated that experimental shear stress, which triggers Ca^2+^ signals in vascular endothelial cells, causes conformational changes in the B2 bradykinin receptor independently of ligand binding [62].

At the moment we do not have a complete explanation for the biphasic nature of the Ca^2+^ response curve of SMD1-CaM complex formation. It is possible that sequential binding of the C-terminal lobe and N-terminal lobes of CaM to SMD1 is responsible for this behavior and that the short nature of GPER’s submembrane domain 1 sequence sets the stage such property to be discerned. In a speculative scheme, at low Ca^2+^ concentrations, such as at rest in cells, CaM’s C-terminal lobe may interact with SMD1, causing a small conformational change in this domain; as free Ca^2+^ increases, such as during SR Ca^2+^ release or Ca^2+^ entry, the N-terminal lobe may now contribute to the interaction, causing a much larger conformational change. Consistent with this possibility, Ca^2+^ binding to separate N- and C-terminal halves of CaM in the presence of a peptide from the CaM-binding domain of the plasma membrane Ca^2+^-ATPase has been shown to be of biphasic nature [63]. These possibilities represent interesting potential for future studies to delineate the biochemical basis for interactions between CaM and SMD1 and their roles in GPER function, especially at basal conditions in cells.

In summary, we have used a novel approach to identify four CaM-binding domains in GPER and characterize properties of their interactions with CaM. Our data demonstrate that this approach can be used with ease to locate CaM binding sequences in GPCRs in particular and perhaps in other proteins in general. The results of our study suggest that CaM may play a regulatory role in actions of estrogen mediated by GPER. It is interesting to note that the nuclear estrogen receptor ERα has been demonstrated to be a Ca^2+^-dependent CaM-binding protein, whose interaction with CaM stabilizes its dimerization, modulates the interaction with estrogen responsive element and activates transcription [64]–[69]. Together with these earlier findings, the presence of multiple CaM-binding domains in GPER suggests that CaM is important for estrogen signaling at multiple levels. With the large differences in affinity and Ca^2+^ sensitivity, the effects of CaM binding to each of the sub-membrane domains on GPER’s functions present an interesting and necessary area of investigations.
